# Climate policy conflict in the U.S. states: a critical review and way forward

**DOI:** 10.1007/s10584-022-03319-w

**Published:** 2022-02-16

**Authors:** Joshua A. Basseches, Rebecca Bromley-Trujillo, Maxwell T. Boykoff, Trevor Culhane, Galen Hall, Noel Healy, David J. Hess, David Hsu, Rachel M. Krause, Harland Prechel, J. Timmons Roberts, Jennie C. Stephens

**Affiliations:** 1 University of Michigan, Ann Arbor, USA; 2 Christopher Newport University, Newport News, USA; 3 University of Colorado, Boulder, USA; 4 Brown University, Providence, USA; 5 Salem State University, Salem, USA; 6 Vanderbilt University, Nashville, USA; 7 Massachusetts Institute of Technology, Cambridge, USA; 8 University of Kansas, Lawrence, USA; 9 Texas A&M University, College Station, USA; 10 Northeastern University, Boston, USA

**Keywords:** U.S. climate change policy, Climate policy obstruction, State politics, Renewable energy

## Abstract

Many U.S. states have taken significant action on climate change in recent years, demonstrating their commitment despite federal policy gridlock and rollbacks. Yet, there is still much we do not know about the agents, discourses, and strategies of those seeking to delay or obstruct state-level climate action. We first ask, *what are the obstacles to strong and effective climate policy within U.S. states?* We review the political structures and interest groups that slow action, and we examine emerging tensions between climate justice and the technocratic and/or market-oriented approaches traditionally taken by many mainstream environmental groups. Second, *what are potential solutions for overcoming these obstacles?* We suggest strategies for overcoming opposition to climate action that may advance more effective and inclusive state policy, focusing on political strategies, media framing, collaboration, and leveraging the efforts of ambitious local governments.

## Introduction

Powerful interests have rebuffed climate policy efforts in the U.S., leading to decades of federal government inaction and heightened attention at the state level, where there has been comparative progress (Rabe 2007; Bromley-Trujillo et al. 2016). A great deal has been written about this shift to the states, and a robust literature on U.S. climate federalism has emerged (e.g., Karapin 2016; Rabe 2011; Thomson 2014; Woods 2021), including the significant climate policy action undertaken by states in the context of federal gridlock and policy rollbacks (Bromley-Trujillo and Holman 2020). For example, after President Trump announced U.S. withdrawal from the Paris climate agreement, cities and states formed coalitions with major companies and institutions to proclaim, “We Are Still In” (We are still in 2021). Twenty-five governors joined the United States Climate Alliance (USCA), committing their states to the goals of the Paris Agreement (USCA 2019).

Although many states have adopted climate policies, there remain significant obstacles to passing *strong and effective* state-level climate policies rather than merely symbolic policies that set goals without mandates or that do not include penalties for noncompliance (Stokes 2020). Even in liberal states without significant fossil fuel production, policy efforts often fail to meet their emission reduction targets (Basseches 2019; Culhane et al. 2021). While there has been a proliferation of research on state-level climate and energy policy since the mid-2000s, scholarship using politics as an organizing, theoretical frame has only exploded in the last few years, making a synthesis geared toward this question of political obstacles quite timely (Woods 2021). This review thus focuses on two core questions:

*First, what are the obstacles to adopting robust climate policy within U.S. states?* We review the political structures and interest groups that slow or dilute action, and we also examine emerging tensions between climate justice and the more market-oriented approaches traditionally taken by many mainstream environmental groups. Furthermore, we explore the ways that conservative countermovements have shaped public opinion and elite decision-making on climate policy.

*Second, what are potential solutions for overcoming these obstacles?* Rather than ending with a mere summation and call for more research, we distill some strategies for overcoming opposition to climate action that may advance more effective and inclusive state policy. We suggest strategies to advance ambitious solutions, with a focus on political strategies, media framing, collaboration, and leveraging the efforts of ambitious local governments.

This review is structured in three main sections: (1) an overview of state climate policy efforts, (2) obstacles to robust state-level climate mitigation policy, and (3) solutions to maximize state-level climate policy effectiveness. Although our focus is entirely on the U.S., many of the obstacles and strategies for overcoming them are not unique to the U.S., and this review is likely to be relevant for researchers, policymakers, and advocates in other countries and at other levels of government. We begin with a brief overview of state climate policy efforts before moving to our discussion of obstacles and solutions.

## An overview of state climate efforts

The focus of this paper is on climate mitigation policy, which can take many forms including broad-based climate policies, transportation policies, and electricity sector policies that have climate change implications (Grant et al. 2014; Bromley-Trujillo and Holman 2020). In the U.S., states have led in this area since the early 2000s as detailed in scholarly work (e.g., Rabe 2004; Matisoff and Edwards 2014; Bromley-Trujillo and Holman 2020).

These studies demonstrate a wide range of policy activity that centers on broad-based climate change efforts such as climate action plans, carbon cap-and-trade, and GHG reduction targets, transportation sector policies including low carbon and alternative fuel standards, and electricity sector policies such as renewable portfolio standards, net metering, and decoupling.

While it would be impossible to discuss in detail every policy states have adopted here, we begin by presenting an overview of key policy instruments states have used with an emphasis on the more frequently adopted policies across the aforementioned categories (broad-based climate efforts, transportation sector and electricity sector policies). Table 1 gives a description of state climate policy instruments, as identified by the Center for Climate and Energy Solutions, which emphasize some of the more comprehensive state climate policies to date.

**Table 1:** State climate policy innovations

Policy	Description
Low Carbon and Alternative Fuel Standards	Requires transportation fuel to contain a minimum amount of renewable fuels, such as cellulosic biofuel
State GHG Emissions Targets	Requires the state to reduce GHG emissions by a specified amount at a specific date
Carbon Cap and Trade	A program that sets a cap on CO_2_ emissions and creates a marketplace for buying and selling credits
Renewable Portfolio or Clean Energy Standards (RPS/CES)	Requires a specified percentage of a state’s electricity to be sourced from renewable energy by a specified date
Decoupling	Power regulation adjustments that sever the link between energy sold and revenue
Climate Action Plans	A strategic plan that provides a blueprint for climate mitigation and adaptation in the state

Source: Center for Climate and Energy Solutions

**Fig. 1:**
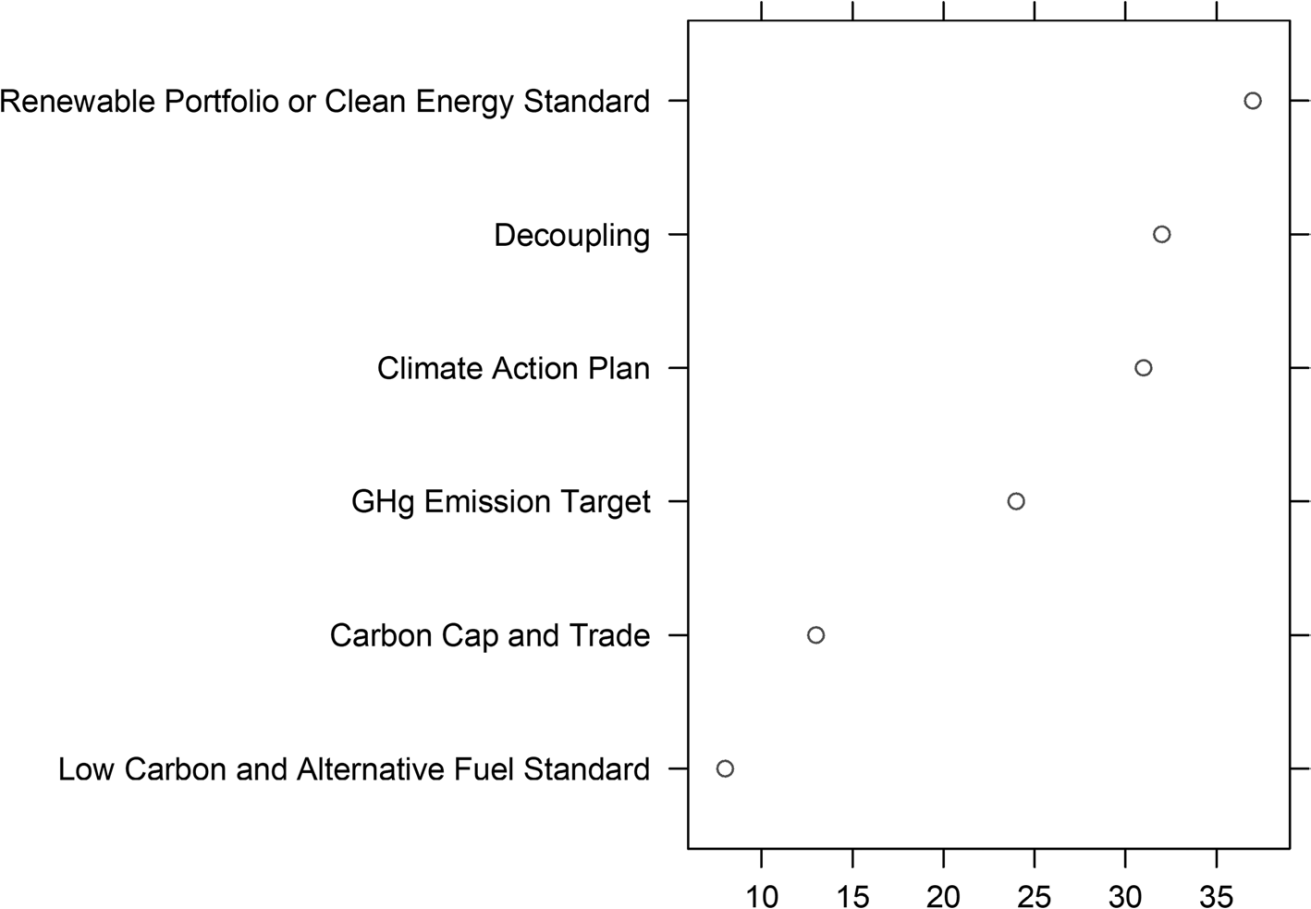
Key climate policy enactments across states by 2021

Figure 1 shows the frequency of these policy adoptions by 2021, demonstrating considerable variance in total adoptions.

These policies are not the only efforts states engage in. For example, when it comes to the electricity sector and energy efficiency, 20 states have enacted a green building standard requiring public buildings to meet LEED or related standards (DSIRE 2021; May and Koski 2007). Another 15 states have adopted an appliance efficiency standard that goes beyond federal requirements. With regard to transportation, 45 states have adopted some form of incentive for hybrid/electric vehicles to date (Hartman and Shields 2021).

Across state legislatures in 2020, policy has centered on environmental justice and equity bills, development of electric vehicle infrastructure, and electrification of the transportation sector through tax incentives (Andersen et al. 2021). Despite these significant advances, it is clear that state policy actions are highly variable and currently insufficient to meet U.S. climate mitigation goals. Variability is evident when looking at RPS policies, which have been adopted by 37 states with considerable differences in stringency. For instance, South Carolina has a modest requirement of 2% generation capacity from renewable energy by 2021, compared to California, which requires 100% of electricity from renewable sources by 2045. Moreover, several states have engaged in policy retrenchment in recent years by making reductions to their state RPS targets (e.g., Ohio) or adjusting their net metering programs through phase outs, or the introduction of fees (e.g., Kentucky, Indiana) (Bromley-Trujillo and Holman 2020). Absent more consistent and stringent state policy coverage, the U.S. cannot meet climate mitigation objectives, necessitating efforts to reduce obstacles to more robust state climate policy activity.

## Obstacles to subnational climate policy

In this section, we discuss the obstacles to more robust and widespread state-level climate policy. We examine four obstacle categories: (1) governance and institutions, (2) media and public opinion, (3) industry and interest group opposition, and (4) divided pro-climate coalitions.

### Governance and institutions

Political party governance and institutional arrangements in state government are important obstacles to climate policy action, particularly as environmental issues have become more politically polarized over time (Daniels et al. 2012). Democratic control of state governments facilitates climate policy adoption while Republican leadership acts as a veto point for climate legislation, often necessitating a Democrat trifecta to achieve bill passage (Bromley-Trujillo et al. 2016; Coley & Hess 2012; Trachtman 2020). There is also evidence to suggest a “counter-partisan response” at the state level (Miras and Rouse 2021); that is, when one party controls the federal government, the opposing party may become emboldened to act at the state-level (Bromley-Trujillo and Holman 2020).

State institutional configurations such as legislative professionalism and administrative capacity also play an important role. Legislative professionalism, which refers to variation in time in session, salary, and staff in state legislatures (Squire 2007), can play a meaningful role in the quality and quantity of policy adopted by state governments. For climate change, it is particularly important because this issue is technical and complex. Professionalized legislatures tend to be more adept at crafting innovative legislation around complex issues, while refuting anti-climate “model legislation” from groups like the American Legislative Exchange Council (ALEC), a conservative-business alliance known for providing anti-climate legislation for state legislators to formally introduce (Hertel-Fernandez 2014; Jansa et al. 2019).

Research also shows that the organization of the executive branch has an important effect on policy outcomes (Karapin 2016; Raymond 2016). To illustrate, Carlson (2017) demonstrates that administrative/regulatory capacity has been key to California’s climate policy innovation. Meckling and Nahm (2018) argue that when state legislatures delegate significant policymaking authority to executive branch agencies, the latter tend to be relatively depoliticized and less susceptible to powerful interest groups. However, the success of administrative delegation is contingent on administrative capacity (Meckling and Nahm 2018).

Another important institutional consideration is the formal powers afforded to majority party leaders and committee chairs in legislative bodies (e.g., Anzia and Jackman 2013; Anderson et al. 2016). Formal powers are in part a product of other institutional arrangements, such as the presence or absence of term limits (Carey et al. 2006; Mooney 2012; Shay 2020). Basseches (2019) shows that the concentration of institutional power in the hands of majority party leadership, even when the majority party is Democratic, facilitates access and influence for business actors while limiting it for environmental groups.

### Media and public opinion

Media coverage and public opinion around climate change also present obstacles to robust climate policy in the case where public concern is low (Bromley-Trujillo and Poe 2020; Bromley-Trujillo et al. 2019) and when media coverage frequency and content fail to raise the issues’ salience (Boykoff et al. 2021).

Media representations are powerful conduits of climate science and policy (mis)information. Moreover, media coverage of climate change, which is heavily driven by elite cues, is likely to shape public attitudes (Carmichael and Brulle 2016). Research on media portrayals of science-based issues shows that quantity and content of media coverage influences state-level agenda-setting (Bromley-Trujillo and Karch 2019). As such, when coverage presents climate science as uncertain, or fails to engage the views of different subgroups (Howarth and Black 2015), that coverage can shift climate change off of public and governmental agendas (Boykoff et al. 2021).

Public opinion also emerges as a barrier to climate action through influence on state legislative agendas (Bromley-Trujillo et al. 2019) and broader public discourse. Despite the scientific consensus on climate change (IPCC 2014), public attitudes are highly polarized (Guber 2013; McCright and Dunlap 2011). Variation in climate attitudes tends to fall in four primary areas: public understanding and awareness, the existence of climate change, issue salience, and public policy (Egan and Mullin 2017). On understanding and awareness, a 2020 Yale survey showed that only a slight majority (55%) of the public believes that “most scientists think global warming is happening,” which does not reflect the current scientific consensus (Leiserowitz et al. 2020; Egan and Mullin 2017). Furthermore, while a large majority of the public (72%) say climate change is happening, only a smaller majority (57%) indicate that it is human-caused (Marlon et al. 2020).

With respect to issue salience (i.e., the level of importance placed on climate change), U.S. residents have historically seen climate change as a low governmental priority (McCarthy 2016), especially compared to the populaces of other countries (Egan and Mullin 2017). Attitudes toward specific climate policies are mixed, and sensitive to question wording. Support tends to be high for renewable energy investment and broad climate policy pronouncements (Bowman et al. 2016; Stoutenborough et al. 2014), but lower for more complex policies and for those imposing costs (Stokes and Warshaw 2017).

Partisan differences are also significant barriers to climate policy action. Republicans are more likely to believe that climate change does not exist, is the result of natural processes, or is too costly to address (Hornsey et al. 2016). Additionally, factors shown to influence climate attitudes (e.g., extreme weather experience and scientific knowledge) are moderated by partisanship (Shao et al. 2017). Direct experience with extreme weather is perceived differently by Republicans, Independents, and Democrats, with Republicans typically understating the seriousness of their experiences, and Independents most sharply swinging with recent weather (Hamilton 2011; Hamilton and Stampone 2013; Shao et al. 2017; Myers et al. 2012).

### Industry and interest group opposition

A third source of climate policy obstacles are interest groups, including fossil fuel and business lobbies, electric utilities, and a broad conservative countermovement.

#### Fossil fuel lobbying, corporate political activity, and corporate-state relations

U.S. federalism delegates immense authority to states when it comes to climate and energy policy, and state efforts have expanded in the face of federal inaction (Karapin 2020; Thomson 2014; Rabe 2011). This creates new opportunities for corporations and their lobbyists to influence climate policy. Initially, the increased authority of states prompted researchers to anticipate a “race to the top” with some states setting higher environmental standards (Fiorino 2006). However, subsequent research showed that the political economy of the environment often generates a “race to the bottom,” with some states competing for fossil fuel companies to develop their energy resources (Rabe 2007, 2013; Davis 2012; Cook 2017). Furthermore, after states become dependent on employment and tax revenues from the fossil fuel companies, they tend to make concessions to them. Wingfield and Marcus (2007) show that many of the states most dependent on fossil fuel industries have among the weakest environmental policies (e.g., Wyoming, Alabama, North Dakota, West Virginia, Louisiana).

The political alignment of subnational states and the fossil fuel sector is also motivated by economic co-dependence between state governments and the fossil fuel sector, resulting in states’ protecting business interests in order to advance the states’ economic growth and development agendas. However, this strategy can create conflict with neighboring states where air quality is adversely affected by high-polluting states. To mediate this conflict between states, the Obama Administration enacted the Cross-State Air Pollution Rule to limit the drift of airborne pollution across state borders. This policy quickly became a contested terrain between states and the federal government over jurisdiction, and it was resolved by the federal government making concessions to high-polluting states (Prechel 2012). Economic co-dependence also results in other actions by states that benefit the fossil fuel industry. To illustrate, several Republican lawmakers in Texas recently proposed legislation that threatened to divest the state’s more than $100 billion in retirement funds from banks and asset managers that boycott the fossil fuel sector (Douglas 2021).

Further, relaxed antitrust enforcement at the federal level has permitted the emergence of giant fossil fuel corporations (e.g., ExxonMobil, Koch Industries), which have virtually unlimited capital to spend on lobbying, political contributions, and media campaigns to oppose climate legislation. To illustrate, the Koch Brothers spent some of their $80 billion in wealth on an extensive media campaign to discredit scientific research on environmental pollution (Mayer 2017). Furthermore, during the 2019–2020 federal election cycle, the Koch Brothers’ Super PAC, Americans for Prosperity Action, spent more than $47.7 million on federal elections in disclosed contributions compared to less than $41.5 million for all contributions by the largest 20 environmental organizations (Open Secrets 2020a, 2020b). Moreover, historically, Americans for Prosperity Action has spent much more on undisclosed contributions (i.e., dark money), which reached $407 million during the 2012 federal election (Fang 2014).

Some of the most active anti-climate policy trade groups include state chapters of the American Petroleum Institute, the Oil Heat Institute, and associations of manufacturers and state Chambers of Commerce. Trade organizations are often dominated by a few of the largest firms, which have key positions on boards of directors, experts to serve on policy-drafting committees, and influence over hiring in state governments. Interviews with Chamber of Commerce representatives and observations of testimony show substantial variation in major industry group positions, though they generally resist new taxes or regulations (Culhane et al. 2021).

Despite their massive resources, fossil fuel corporations and trade groups do not have the expertise to address every environmental issue. Thus, many are members of the neoliberal policy organization, ALEC, which is committed to small government and unregulated markets. ALEC is dominated by the largest corporations because it charges high membership dues in exchange for model legislation that it distributes to state lawmakers. ALEC also operates as a networking mechanism that facilitates connections between corporations with shared interests (Prechel 2021a). For example, Koch Industries created a political coalition with the former Enron Corp. and succeeded in enacting model legislation in twenty-four U.S. states (Hertel-Fernandez 2019).

#### Utilities

Given that electricity accounts for more than a quarter of U.S. greenhouse gas emissions (U.S. EPA 2018), electric utilities are critical actors in state-level climate policymaking (Prechel 2012; Basseches 2020; Isser 2015; Stokes 2020). The U.S. electric sector is complex, with variation across states in the degree to which utilities are private corporations (known as “investor-owned utilities”) or customer-owned utilities, which can either be government-owned or electricity cooperatives (Greenberg & McKendry 2021). However, most U.S. residents receive electricity from investor-owned utilities (IOUs) rather than from public or cooperative organizations (U.S. Energy Information Administration 2017). States vary in the degree to which they undertook efforts to break up vertically integrated utilities and introduce retail competition beginning in the late 1990s (Borenstein and Bushnell 2015), and this variation led to differences in how these actors came to view climate policy proposals (Basseches 2020).

The technical complexities of utilities’ operations and regulations make the policy area less accessible to many observers, but the scholarship that attends to IOUs' political activities shows them to be among the most politically powerful actors in state-level climate policymaking (e.g., Basseches 2020; Culhane et al. 2021; Stokes 2020). The sources of their influence include monopoly control of electricity distribution, unparalleled technical expertise, their lobbying force, and flexible corporate organization (Basseches 2020). The latter has facilitated mergers and acquisitions that have allowed utility parent companies to operate across state lines, despite being mainly regulated at the state level (Hempling 2020; Prechel 2021a, b).

Despite their political power, the degree to which utilities undermine climate policy is unclear. The primary concern of IOUs is to maximize shareholder profits, but because of the manner in which they are regulated, state-level climate and renewable electricity laws do not necessarily contradict this goal (Basseches 2020). In fact, Basseches (2020) finds IOUs have been instrumental actors in *supporting* ambitious RPS policies in states such as California, Massachusetts, and Oregon. However, other utilities have historically obstructed or slowed climate policy progress, often mobilizing quietly to achieve these objectives. Whether they serve as proponents or obstructionists depends on their fuel mix, the individual state-level policy regime and the particular policy at hand; for example, utilities tend to uniformly oppose solar net metering policies because they threaten their monopoly control of the electric grid (Stokes 2020). As Romankiewicz et al. (2021) find, the largest utilities set renewable portfolio goals but then fail to make the investment decisions necessary to achieve them. They also find that the preexisting portfolios of utilities (prior to the adoption of climate policy) is typically the strongest predictor of future investment decisions.

An important debate has emerged about whether IOUs versus public- or customer-owned utilities are preferable for advancing climate policy (Brown & Hess 2016; Homsy 2020; Heiman & Soloman 2004). From the standpoint of “energy democracy” (Greenberg & McKendry 2021), public power is clearly preferable. However, when it comes to renewable portfolios, public power’s track record is less clear (Romankiewicz et al. 2021). Although more research is needed to further specify conditions for utilities’ *support* of effective climate policies, it is clear that utilities are a powerful source of obstruction in many cases. For example, at the enforcement and implementation stages, utilities often dominate public utility/service commission rulings (e.g., Stokes 2020).

#### Conservative countermovement

Many of the aforementioned industry groups have also been central players in a broad countermovement that opposes the scientific community and the climate movement’s push for action (Brulle 2020; Dunlap and McCright 2010; 2015). This countermovement has been a significant contributor to climate policy obstruction (McCright and Dunlap 2003). Climate change narratives have frequently been coopted by the fossil fuel sector, conservative politicians and think tanks, media, and interest groups. All of these actors comprise a climate denial movement that, at times, coordinates their efforts.

The beginnings of the climate denial movement emerged in response to the environmental movement’s success in passing major legislation such as the Clean Air Act in the 1960s-1970s. Soon after, the Reagan administration took direct aim at environmental regulations under a neoliberal mantra of free markets. These actions in turn prompted a swift backlash from the environmental movement (Brulle 2020). Those opposed to environmental regulations learned an important lesson from this backlash; rather than directly attacking environmental programs, efforts should instead focus on undermining the science that supports such policies (Jacques et al. 2008; Michaels 2008). The conservative countermovement has constructed three primary narratives about climate change: (1) that it does not exist, (2) that if it does exist, it is not anthropogenic, and is possibly even desirable, and (3) that any efforts to mitigate climate change would harm the economy (Dunlap and McCright 2010).

The climate denial movement is financially supported by the fossil fuel industry and other conservative businesses and foundations (McCright and Dunlap 2003). These funds flow to conservative think tanks that elevate contrarian scientists casting doubt on the veracity of anthropogenic climate change. Parts of the movement organize campaigns to create uncertainty around climate modeling, methodology, and the integrity of scientists themselves (Hess 2014). One of the first such climate denial think tanks was the George C. Marshall Institute (Oreskes and Conway 2008). Others include the Cato Institute, the Competitive Enterprise Institute, the Heritage Foundation, and the Heartland Institute. Conservative think tanks and foundations brand themselves as an alternative universe of scientists outside of academia. They publish policy briefs, books, and analyses that question the credibility of climate science (McCright and Dunlap 2015).

Although the scientists associated with these think tanks often lack relevant credentials, their findings are amplified by Republican politicians (Dunlap and Jacques 2013). Contrarian scientists are disproportionately vocal and present at congressional hearings. Republican politicians typically refer to climate change as a hoax and have invoked cold weather and “Climategate” to signal that the science is corrupt (Jacques et al. 2008).

Think tank reports are also amplified by conservative media including radio hosts (Wolcott 2007), the Wall Street Journal, Fox News, and columnists such as George Will (Boykoff 2013; McCright et al. 2016). Media coverage on climate change, in turn, likely influences elected officials (Bromley-Trujillo and Karch 2019) and also polarizes public and elite attitudes (Leiserowitz et al. 2020; Tesler 2018).

Although scholarship often focuses on the climate denial movement’s influence on national politics, the movement is closely linked to efforts to sway state-level politics. The climate denial movement aligns with the State Policy Network, Americans for Prosperity, and ALEC, which often work in concert to stall state-level policy (Hertel-Fernandez 2014, 2019). Conservative foundations (Brulle 2014; Farrell 2019) as well as personnel links (Farrell 2016) connect these organizations in a centralized network.

### Divided pro-climate policy coalitions

One obstacle to subnational climate policy that is perhaps less well recognized is the fragmentation of pro-climate policy coalitions. One source of fragmentation is divisions among the different alternative or renewable energy industries, which must operate in a political arena dominated by powerful fossil fuel incumbents (Kelsey and Meckling 2018). For example, a study of lobbying and testimony in Massachusetts found that more concentrated renewable energy industries were better able to engage in paid lobbying than dispersed ones (Culhane et al. 2021). Relatedly, Si and Stephens (2021) find disparate participation among solar developers and installers surrounding efforts to target solar installation among low-income households in Massachusetts. The solar industry is more fragmented in small installation firms, whereas the wind industry has higher capital barriers to entry and is consequently concentrated in a few, large firms. Solar firms are further divided between rooftop residential developers and those installing utility-scale projects, and between in-state and out-of-state firms (Stokes 2020).

In addition to divisions based on concentration, size, and capacity to influence politics/policy, the renewable energy industries also tend to restrict their participation to issues that affect them most directly. For example, studies in Massachusetts and Rhode Island revealed that solar, wind, and other renewable firms did not show up to testify for legislation (e.g., carbon pricing) that did not target benefits to their economic sector. By contrast, environmentalists testified in large numbers in favor of the full range of climate bills. The picture that emerges is a fragmented renewables sector, with firms only lobbying and testifying for their own, narrow issues and sometimes battling each other over carve-outs for particular technologies in state-level RPS policies (Culhane et al. 2021).

Another source of division in pro-climate coalitions is between those who advocate for market-based, technocratic approaches to climate mitigation versus those who advocate for more holistic, climate justice approaches involving large public investments in jobs, infrastructure, equity, and health (Boyle et al. 2021). The more holistic approach acknowledges the power of the polluting elite, who have strategically invested for decades in undermining public trust in government and minimizing protections and support for marginalized communities, communities of color, and economically disadvantaged groups who are being disproportionately impacted by climate change and pollution (Stephens 2020). To further concentrate their wealth and power, big business has also reduced worker rights and protections, and it has shifted corporate culture to prioritize shareholders instead of workers (Stephens 2020). This approach tends to be aligned with progressive-left political coalitions, whereas the technocratic approach has a more moderate political position and tends not to emphasize issues of structural inequality. The structural vulnerabilities and under-investment that has been revealed by the COVID-19 pandemic have strengthened the political appeal of the holistic investment-based climate justice approaches (Boyle et al. 2021).

Most adopted and proposed state-level climate policies are based on a narrow, technocratic, carbon-centric model, which misses opportunities to invest in marginalized communities (Galvin and Healy 2020). To date, climate policy has been largely designed within the context of “climate isolationism,” which refers to the common framing of climate change as a narrow, isolated, discrete, scientific problem that requires a technological solution (Stephens 2020). Decision-makers working through a climate isolationism lens often focus in a technocratic way on achieving carbon reductions while inadvertently dismissing the social justice implications and human dimensions of these measures (Stephens Forthcoming 2021). Controversy surrounding California’s cap-and-trade program illustrates the conflict between climate justice and mainstream, technocratic policies (Basseches et al. 2021).

Until the Green New Deal framework gained traction on the national stage in 2018 (Galvin and Healy 2020), climate policies were often limited to market-based approaches. With more diverse leadership, including women, people of color and Indigenous people, a new approach is emerging that links climate/energy policy with jobs and economic justice, health, food, housing, and transportation. Several states and cities have proposed ambitious Green New Deal policies, such as New York’s Climate Leadership and Community Protection Act (Boyle et al. 2021). This approach focuses on justice-oriented policies and direct investments in under-invested in households and communities. For example, climate justice proponents are now pushing for more equitable housing and community development, equitable access to clean and affordable energy, and more inclusive public engagement around climate policy development (Clifton and Kelly 2020). An expansion of the “just transition” concept includes worker protections and recognition of fossil-dependent communities and consumers (Healy and Barry 2017).

A related division in pro-climate policy coalitions is between actors who advocate for energy-transition policies (including those with a justice orientation) and actors who focus more on opposition to unwanted energy infrastructure and fossil fuel reliance. A review of many different types of state-level climate policies revealed that there are many more policies to advance renewables than there are to end fossil fuel reliance (Burke and Stephens 2017). Climate justice activists have thus engaged in multi-year protests targeting fossil fuel infrastructure, advocating for supply-side climate policies such as fracking bans, fossil fuel moratoria, state pension divestment campaigns, and litigation for climate harms (Piggot 2018; Healy & Barry 2017). Controversy about whether or not institutions and investment portfolios should “divest” from fossil fuels demonstrates this division in pro-climate policy coalitions (Trinks et al 2018); many colleges and universities have resisted the urge to divest and have pledged instead to “invest” in renewables (Mikkelson et al., 2021, Stephens et al 2018).

Another important division in pro-climate coalitions is between the labor and environmental wings of progressive coalitions. Since the 1990s, “green jobs” and economic development frames have emerged along with some partnerships between unions and environmentalists (e.g., the United Steelworkers and the Sierra Club in the BlueGreen Alliance, (Hess 2012). These partnerships have reduced the longstanding image of environmental policy as a threat to working-class jobs; however, not all unions support the “green jobs” approach, and mistrust and opposition remain. For example, in some states, utilities have worked closely with their own workers and unions to mobilize opposition to energy-transition policies and fossil-fuel opposition (e.g., anti-pipeline mobilizations) by arguing that the opposition policies will harm local economies, taking away jobs. In states with a strong extractive fossil-fuel sector, anti-green labor alliances can also extend beyond utility unions to unions and other workers in the mining, drilling, and processing industries.

## Solutions to advancing robust climate policy

Despite the challenges just discussed, there are promising strategies for moving robust state-level climate policies forward that we cover below.

### Governance and institutions

A significant barrier to climate action centers on governance around a highly polarized policy issue. As such, the first set of solutions concerns electoral strategies and working with local governments to move climate mitigation policy forward in the states.

To begin, elections matter, and the need to elect political leaders motivated to address climate mitigation is essential. The impacts of the Sunrise Movement and other progressive groups on U.S. federal and state elections in 2018 and 2020 showed that pro-climate positions and policies can quickly become influential, at least in the Democratic Party (Stuart et al. 2020). However, it will take large majorities of climate policy advocates to influence or replace legislative leadership in state governments. Furthermore, Basseches (2019)’s findings suggest that even in states with overwhelming Democratic majorities, strong climate policy can be elusive; elected climate champions must be elevated to positions of institutional power within the majority party caucus (e.g., Speaker of the House, Senate President, etc.).

An improved political strategy is needed, including climate advocates’ engagement in primary (as well as general) elections. Unfortunately, most non-profits working in this area are 501(c)3 organizations, which are constrained from lobbying and endorsing political candidates by U.S. tax laws (IRS 2021). Philanthropic foundations and the NGOs they fund tend to be extremely cautious about political action, and this makes many of them less effective (Berry 2003). Despite this, these groups fill a special need because they can undertake efforts like distributing questionnaires to candidates, interviewing them for endorsements, electioneering, and forming political action committees.

In the many states where electing climate advocates proves to be difficult, there are also ways to encourage a path to renewable energy as a source of economic development and growth (Carley and Lawrence 2014). Policy instruments like clean energy and renewable portfolio standards can be discussed in terms of economic development, which may encourage conservative state governments to act (Carley and Lawrence 2014). For instance, Texas was an early adopter of a modest RPS (compared to today’s standards) that yielded significant early gains in wind energy development that also facilitated economic growth (Slattery et al. 2011).

Moreover, innovative local governments still have opportunities to act when state leadership chooses not to. Local governments can reduce GHG emissions by adopting policies that promote/require clean and efficient energy use. They can also influence state governments via formal lobbying efforts or, indirectly, by demonstrating innovative approaches that can be scaled-up. In the U.S. and globally, trans-municipal climate and sustainability networks—including C40 Cities, ICLEI, and the Urban Sustainability Directors Network—advance these avenues and have been credited with shaping the landscape around local governments’ climate policy engagement (Acuto 2016; Nguyen Long and Krause 2020).

Many local governments in the U.S. go beyond federal and state climate change policy (Hughes 2019; Krause and Hawkins 2021). After then-President Trump announced the U.S.’ withdrawal, over 290 municipalities committed to honor the Paris Climate Agreement (We Are Still In 2021), and by 2021, over 150 pledged a transition to 100% renewable energy (Sierra Club 2021). Local governments can shape energy use practices within their own operations and often have authority over building codes, public transportation, waste management, and a variety of land use and infrastructure decisions impacting GHG emissions. The aggregate impact of local efforts is potentially large; however, debate persists around the magnitude of their impact, and sustained progress by local governments has been highly uneven (Gurney et al. 2021; van der Heijden et al. 2019).

Municipalities frequently lobby higher-level governments to pass policies that yield local benefit (Goldstein and You 2017). Regarding climate change, three strands of local lobbying efforts are evident. First, municipal lobbying is most often aimed at acquiring money and resources—as illustrated by the coordinated efforts advocating the inclusion of Energy Efficiency and Conservation Block Grants (EECBG) in the 2009 American Reinvestment and Recovery Act (US Conference of Mayors 2014). Second, local governments may lobby their state governments for the expanded authority necessary to enact specific portions of their climate action plans (Hughes 2019). Finally, local governments and trans-municipal networks can seek to persuade higher levels of government to enact their own climate policies (Lee and Jung 2018; Curtis and Acuto 2018). For example, cities may ask their states to develop comprehensive energy plans, and organize efforts to sway international bodies to adopt stricter mitigation commitments.

“Leading from below” is a final way that local governments are impacting broader climate policy. Often credited for their innovative climate programming, these efforts are experiments that can be up-scaled and adopted by state governments (Kern 2019). However, such innovation can be risky when it occurs in conservative states hostile to climate objectives. In these venues, local-state conflict often plays out via state preemption laws, which revoke local authority to act on certain issues or in certain manners (e.g., fracking restrictions and electricity provider choice) that results in stifling local policy innovation (Riverstone-Newell 2017).

### Media and public opinion

Second, media and public attitudes critically shape individual and collective engagement around contemporary climate challenges (Boykoff 2011). As such, solutions to climate policy inaction should pursue efforts to influence the media and public opinion landscape.

As indicated previously, media coverage presents an obstacle and an opportunity to motivate climate action. In order to keep climate policy on state-level agendas, there is a need to maintain high levels of climate change media coverage, even as other crises grab headlines. In addition, the content of that coverage is important. Although the frequency of climate change coverage has increased globally, challenges associated with quantity and quality of representations of climate change topics remain (Boykoff et al. 2021).

Analyses of media representations demonstrate how media portrayals (quantity and quality) play into climate governance at multiple scales in the U.S. (Brulle et al. 2012; Fisher 2013). For example, climate change garnered coverage through stories intersecting *political*, *economic*, *scientific*, *cultural* as well as *ecological* and *meteorological* themes, which ultimately influence public and political discourse on the subject (Boykoff et al. 2021). Media framing of climate change can also affect attitude change and scholars have considered how climate change communication must be tailored to different audiences to be persuasive. Most prominently among audience segmentation work resides the ‘Global Warming’s Six Americas’ project on climate communication (Leiserowitz et al. 2011). Howarth and Black (2015) note that “the communication of climate change historically has been generic, untailored and untargeted” (p. 506). As such, more effort is needed to carefully frame communications and dialogue that values different perspectives on climate change in order to increase concern and engagement across each of the 50 US states.

In addition to legacy media portrayals, social media platforms play an important role in the public arena (Tandoc and Eng 2017; Fownes et al 2018). Given the potential for social media to drive mainstream media coverage, savvy climate policy advocates can use social media to generate coverage of climate change and craft a message that can move varying subgroups (Anderson 2017).

While media coverage can influence public attitudes, research suggests that attitudes can shift through the following strategies: (1) depoliticizing climate change through alternative issue framing and discussions of policy co-benefits, (2) amplifying current support for climate policies, and (3) raising the salience of climate change through connections with visible climate change impacts.

Although some U.S. residents remain doubtful or dismissive of climate change, research shows that linking the issue to economic development and public health can increase policy support, even among Republicans (Rabe 2004; Stokes and Warshaw 2017). Moreover, “climate policy bundles” that bring together broader issues, like economic inequality and environmental justice, may increase climate policy support (Bergquist et al. 2020).

Though climate policy attitudes vary, several policy options receive considerable public support, including investment in renewable energy, tax rebates, subsidies, and renewable portfolio standards (Stokes and Warshaw 2017; Stoutenborough et al. 2014). Nevertheless, bipartisan public support for addressing climate change has not always translated into action by elected officials. Politicians (particularly Republicans) and their staff tend to drastically underestimate their constituencies’ support for climate policy (Hertel-Fernandez et al. 2019). Consequently, efforts to educate policymakers about existing public support and raise the salience of climate change have the potential to promote policy change.

Despite these opportunities, because climate impacts are presented to the public as complex and abstract, they are perceived to be far away and uncertain, which makes it difficult to raise public awareness (Lubell et al. 2007; Boykoff 2019). However, as climate impacts become more frequent, it may become easier to raise their salience. Some scholars find that temperature anomalies and extreme weather increase climate concern, though effects are temporal (Borick and Rabe 2014; Egan and Mullin 2012; Konisky et al. 2016); others find no link between the two (Brulle et al. 2012; Mildenberger and Leiserowitz 2017). Still, as climate impacts become more prevalent, there may be more opportunities for political actors, the media, and interest groups to educate the public on climate risks and to encourage policy action (Howe et al. 2015).

### Industry and interest group opposition

Third, as a number of powerful industries and other interest groups have moved to obstruct climate policy, there is a need to either leverage or reduce the power that these groups wield over climate mitigation policy.

To begin, IOUs have enormous political power that can be leveraged to *promote* ambitious state-level climate policies. In addition, there are pathways available to reduce their power, if they cannot be won over. Basseches (2020) finds that in states like California and Massachusetts, with restructured electricity sectors in which IOUs no longer own fossil fuel generation, a suite of policies rewarding IOUs financially for promoting energy efficiency can neutralize opposition to economy-wide GHG reduction policies. IOUs have supported ambitious RPS policies in other states, like Oregon, as well (Basseches 2020). States where IOUs support climate policy are likely to be “blue states” (Adua and Clark 2021), consistent with the literature on the role of partisanship in climate policymaking (e.g., Coley and Hess 2012; Fowler and Breen 2013; Vasseur 2014). Unfortunately, this strategy of leveraging IOUs’ political power does not work for net metering policies, which IOUs oppose, even in the blue states (Hess 2016). Still, Smith et al. (2021) suggest that IOUs’ opposition to net metering can be mitigated by policy designs that give utilities credit toward their RPS requirements when their customers install solar panels.

However, some IOUs continue to obstruct state-level climate policy (Stokes 2020). One pathway toward motivating IOUs to change is the use of local-level and private-sector resolutions in support of 100% renewable or clean energy (Greenberg and McKendry 2021; Hess and Gentry 2019). Another pathway is the growth of community-choice aggregation (CCA) in states where it is authorized (Hess and Lee 2020). CCA is easier to achieve than municipalization, which has numerous hurdles (e.g., strong utility resistance, capital cost, and the lack of local expertise). CCA organizations can also opt for high renewable or clean energy mixes that put pressure on utilities to shift their energy mix and long-term goals. Both of these pathways can help to motivate utilities to adopt stronger long-term energy-transition plans. A third pathway is to shift legislative reform to public utilities commissions; when they are not captured by utilities, the commissions can provide a mechanism for stating broad goals and insulating legislators from utility pressure (Brown and Hess 2016). Municipal (publicly owned) utilities and CCAs offer an alternative method of aligning utilities with climate policy through local governments and elected officials. Cities are often more aggressive than states, in turn leading to more aggressive action by municipally-owned utilities and CCAs on climate policy.

Another pathway to weakening obstructionist IOUs’ power is to increase coalition-formation among non-IOU interest groups, as Brown and Hess (2016) found was the key to success in cases in which pro-climate coalitions included not only environmentalists and the renewable energy industry, but also real estate, insurance, or HVAC companies. Finally, given that IOUs are private corporations selling a public service, it may be advantageous—to the degree it’s constitutional—to reduce their access to private politics by, for example, limiting their campaign spending (Brown 2016).

### Reducing divisions in pro-climate policy coalitions

Reducing divisions in pro-climate policy coalitions requires attention to the different types of divisions that were outlined in Sect. 3.4. One way to reduce intra-industry divisions within the clean or renewable-energy sector is to encourage the development of broader industry associations that link together the disparate, reform-oriented actors (e.g., the solar and wind industries, energy efficiency advocates, and those advocating for Community Choice Aggregation (CCA) (Raymond 2016). Although the specific trade associations may continue to pick their battles based on narrower, industry-specific benefits, if they also support broader associations (e.g., green or sustainable business councils in different states), then some of their political resources can be more easily channeled toward broader coalition activity.

A deeper division is between the more technocratic approaches to climate policy and the justice-oriented approaches, which in the U.S. are reflected in tensions between the moderate and progressive wings of the Democratic Party. Moderates in the party, especially in conservative states, might opt to resist the linkage to justice because they are concerned that the justice framing will reduce the likelihood of gaining crucial conservative support in state legislatures. There is a need to think carefully about framing and coalitions that are attuned to the level of government, the issue, and the relative power of different political constituencies. Research on the “red states, green laws” phenomenon (where “red” refers to Republican states) has started to show the types of pro-climate change policies that can gain traction in more conservative locations (Hess et al 2016). Pro-business, pro-energy choice, pro-health (clean air), and pro-economic development frames can work well in this context, but the laws can also have justice implications even if they are not highlighted for political purposes. But even in these conservative states, the more justice-oriented frames may be successful in the more progressive and diverse cities (the blue islands in the red seas). Likewise, anti-pipeline and other anti-infrastructure mobilizations have great potential to utilize co-existing frames that can bridge political divisions (e.g., property rights for rural landowners and sovereignty for Indigenous people, health and safety concerns for communities, and ecological preservation for environmentalists and local recreation industries).

There is more research on the approaches to overcoming the labor-environmental divisions in pro-climate coalitions, and a strong working partnership between labor and climate policy advocates is integral to a rapid transformation of the U.S. to a low-carbon economy (Basseches et al. 2021; Healy and Barry 2017). State-level just transition policies can play a role in broader “build back better” programs in the aftermath of the COVID-19 pandemic.

One example of successful green-jobs legislation at the state level was the 2009 Green Jobs, Green New York law, which directed revenues from the regional cap-and-trade initiative toward job training and energy-efficiency programs for residential and commercial buildings (Lennon 2017; Hess 2018). These initiatives were part of broader calls for “energy democracy” that included unionized, green jobs (Stephens 2019), and they were also the basis for subsequent reform initiatives introduced under the banner of the “Green New Deal” (GND) (Galvin and Healy 2020).

The New York State Climate Leadership and Community Protection Act was passed in 2019 after years of grassroots advocacy by NY Renews—a statewide coalition that included labor unions, economic justice advocates, environmental organizations and other progressive groups (Boyle et al. 2021). This law set a new benchmark for climate ambition, which includes groundbreaking equity provisions (Senate Assembly 2019). State-level GND proposals are emerging as new vehicles for garnering union support for climate policies (Boyle et al. 2021).

For example, GND proposals in California and Massachusetts are forging new coalitions among unions, environmentalists, and social and racial justice advocates (Boyle et al. 2021).

To maximize the yield of this strategy, the GND movement could engage with electricity unions, one of the most unionized industries in the economy and often a sector of unionized labor that opposes energy-transition policies (Huber 2021). Labor, environmental justice, tribal, and community groups need greater involvement in climate-labor policy decision-making, such as the process that led to Colorado’s Office of Just Transition and Washington State’s Initiative 1631. Creating and expanding government rapid response teams in every state will mitigate job displacement and mass layoffs (e.g., the Rapid Response Team in Massachusetts) (Cha et al. 2021). Bridge funding will also be necessary for regions where the public sector is affected by the withdrawal of fossil fuel tax revenues (Cha et al. 2021).

## Conclusion

State-level climate policy has shown great promise in the context of federal obstruction or inaction. Nevertheless, significant obstacles to robust state-level climate policy remain and this review provides a novel synthesis of the literature detailing these barriers. As we note, scholars describe obstacles associated with governance and political institutions, public opinion and media coverage, industry and interest groups, and fragmentation within pro-climate coalitions. What remains less clear from this scholarship is how we can harness this knowledge to formulate *solutions* to policy obstacles; our primary contribution lies here.

Based on the broad, interdisciplinary literature discussed here, we suggest a series of strategies to move climate change policy forward. The politicization of climate change necessitates bringing other groups into the fold of climate policy support. In addition, there is a need for enhanced coordination among climate policy advocates and potential coalition partners and to support electoral gains for climate policy advocates. To achieve these goals, we suggest the following strategies.

First, climate policy advocates should become more skilled in the game of politics, by employing campaign finance strategies, electoral mobilization, and support for existing elected officials who are sympathetic to climate policy as they seek to gain institutional influence (i.e., ascending to leadership positions, etc.). Climate policy opponents have had a great deal more practice and experience doing this, but there is no reason that proponents cannot learn from them and deploy strategic political operations of their own. A related strategy includes “bottom-up” pressure from local governments and municipalities. Second, climate policy proponents should seek to improve the quality and quantity of media coverage, including by tailoring messages to particular audiences and constituencies and continuously linking climate action to co-benefits.

Third, the political power of IOUs can be leveraged in support of strong climate policy if the right conditions and incentives are put in place so that utilities see opportunities for financial growth as a result of these policies. However, in cases where this is not feasible, efforts should be made to reduce their political power, by empowering municipal utilities and CCAs, by building broad coalitions of non-utility business interests, and, when strategic, by shifting the venue of policymaking between the legislative and executive branches. Finally, divisions within the pro-climate coalition should be reduced. This can be achieved through more inclusive policy design that attends to environmental justice issues as well as by encouraging better coordination among “green business” actors, such as renewable energy firms, energy efficiency consultants, green capital, etc.

Although this review moves the research field toward integrated discussion of climate-policy obstacles and solutions, it also has several limitations that could be the basis for future research. One limitation is that both the problems and solutions have a U.S. focus. Although many countries have undertaken restructuring of their electricity systems, each system is unique, and many still have a larger role for public power than in the U.S. Moreover, the polarized political culture characterized by a climate denial machine and heavy influence by wealthy donors and corporations on political outcomes does not necessarily translate well to other countries. Thus, there is a need for additional comparative research on climate policy obstacles and solutions, which will likely reveal topics that are much more salient in other countries.

Moreover, further work is needed in tailoring these solutions to particular states, considering their distinct partisan tendencies, energy economies, media landscapes and government contexts. Nevertheless, the strategies outlined above should be broadly valuable in reducing state-level climate policy obstacles and ensuring comprehensive progress at the state level despite continued uncertainty regarding federal climate policy. In addition, we have suggested ways of tailoring climate messaging by the media and others to make climate policy action more palatable to Republicans. In the context of energy and climate federalism, the states will likely remain key players in the years to come.

## References

[CR1] Acuto M (2016). Give cities a seat at the top table. Nature.

[CR2] Adua L, Clark B (2021). Politics and corporate-sector environmentally significant actions: the effects of political partisanship on U.S. utilities energy efficiency policies”. Rev Policy Res.

[CR3] Andersen, G, Hartman, K, Shea D, Shields L. (2021) 2020-2021 Legislative Energy Trends. https://www.ncsl.org/Portals/1/Documents/energy/2020-2021_Legislative_Energy_Trends_v04_35914.pdf. Accessed 7 Oct 2021

[CR4] Anderson AA (2017). Effects of social media use on climate change opinion. Oxford Research Encyclopedia of Climate Science.

[CR5] Anderson SE, Butler DM, Harbridge L (2016). Legislative institutions as a source of party leaders’ influence. Legis Stud Q.

[CR6] Anzia SF, Jackman MC (2013). Legislative organization and the second face of power: evidence from U.S. state legislatures. Journal of Politics.

[CR7] Bailey I, Compston H (2012) Feeling the heat: the politics of climate policy in rapidly industrializing countries. Palgrave MacMillan, London

[CR8] Banks J, Stephens JC (2020) "Advancing racial justice means ending fossil fuel reliance." DAME

[CR9] Basseches JA, Rubinstein K, Kulaga SM (2021) Coalitions that clash: California’s climate leadership and the perpetuation of environmental inequality. The Politics of Inequality. D. Pettinicchio, Emerald Publishing Limited. 28:23–44

[CR10] Basseches JA (2019). ‘It happened behind closed doors:’ Legislative buffering as an informal mechanism of political mediation. Mobilization: An International Quarterly 24:265–388

[CR11] Basseches JA (2020). Private power in the U.S. states: business interests and the design of state-level climate and renewable energy policies.” PhD Diss., Northwestern University

[CR12] Bergquist P, Mildenberger M, Stokes LC (2020). Combining climate, economic, and social policy builds public support for climate action in the US. Environ Res Lett.

[CR13] Berry JM (2003) A voice for nonprofits. Brookings Institution Press, Washington, D.C.

[CR14] Borenstein S, Bushnell J (2015). The US Electricity Industry After 20 Years of Restructuring. Ann Rev Econom.

[CR15] Borick CP, Rabe BG (2014). ‘Weather or not:’ examining the impact of meteorological conditions on public opinion regarding global warming. Weather, Climate, and Society.

[CR16] Bowman K, O’Neil E, Sims H. 2016. *Polls on the environment, energy, global warming and nuclear power*. AEI Public Opin. Stud., Apr 21. Washington, DC: Am. Enterp. Inst.

[CR17] Boykoff MT (2013). Public enemy no. 1? Understanding media representations of outlier views on climate change. Am Behav Sci.

[CR18] Boykoff M, Church P, Katzung J, Nacu-Schmidt A, Pearman O (2021) *A review of media coverage of climate change and global warming in 2020*, Media and Climate Change Observatory, Center for Science and Technology Policy Research, Cooperative Institute for Research in Environmental Sciences, University of Colorado

[CR19] Boykoff MT (2011) ‘Who speaks for climate?’ Making sense of mass media reporting on climate change, Cambridge University Press, New York

[CR20] Boykoff MT (2019) ‘Creative (Climate) Communications: productive pathways for science, policy and society’ Cambridge University Press*.* pp 302

[CR21] Boyle AD, Leggat G, Morikawa L, Pappas Y, Stephens JC (2021) (2021). Green new deal proposals: comparing emerging transformational climate policies at multiple scales. Energy Res Soc Sci 81:102259

[CR22] Bromley-Trujillo R, Poe J (2020). The importance of salience: public opinion and state policy action on climate change. J Publ Policy.

[CR23] Bromley-Trujillo R, Butler JS, Poe J, Davis W (2016). The spreading of innovation: state adoptions of energy and climate change policy”. Rev Policy Res.

[CR24] Bromley-Trujillo R, Holman M, Sandoval A (2019). Hot districts, cool legislation: evaluating agenda setting in climate change bill sponsorship in U.S. states. State Polit Policy Q.

[CR25] Bromley-Trujillo R, Holman MR (2020). “Climate change policymaking in the states: a view at 2020.” Publius: The Journal of Federalism 50:446–472

[CR26] Bromley‐Trujillo R, Karch A (2019). Salience, scientific uncertainty, and the agenda‐setting power of science. Policy Studies Journal

[CR27] Brown KP, Hess DJ (2016). Pathways to policy: partisanship and bipartisanship in renewable energy policy”. Env Polit.

[CR28] Brown KP (2016). In the pocket: Energy regulation, industry capture, and campaign spending. Sustain: Sci Pract Policy 12:1–15

[CR29] Brulle RJ (2014). Institutionalizing delay: foundation funding and the creation of US climate change counter-movement organizations. Clim Change.

[CR30] Brulle RJ, Carmichael J, Jenkins JC (2012). Shifting public opinion on climate change: an empirical assessment of factors influencing concern over climate change in the US, 2002–2010. Clim Change.

[CR31] Brulle RJ (2020). Denialism: organized opposition to climate change action in the United States. In Handbook of U.S. Environmental Policy. Elgar Publishing, Cheltenham, UK

[CR32] Burke MJ, Stephens JC (2017). Energy democracy: goals and policy instruments for sociotechnical transitions. Energy Res Soc Sci.

[CR33] Carey JM, Niemi RG, Powell LW, Moncrief GF (2006). The effects of term limits on state legislatures. Legis Stud Q.

[CR34] Carley, S, Lawrence S (2014) Energy-based economic development: how clean energy can drive development and stimulate economic growth. Germany: Springer London

[CR35] Carlson AE (2017). Regulatory capacity and state environmental leadership: California’s climate policy. Fordham Envtl L Rev.

[CR36] Carmichael, Jason T. And Robert J. Brulle (2016) Elite cues, media coverage, and public concern: an integrated path analysis of public opinion on climate change, 2001–2013. Env Polit. 10.1080/09644016.2016.1263433

[CR37] Cha JM, Price V, Stevis D, Vachon TE, Brescia-Weiler M (2021) Workers and communities in transition: Report of the Just Transition Listening Project. https://www.labor4sustainability.org/files/JTLP_report2021.pd. Accessed 18 Jun 2021

[CR38] Clifton R, Kelly C (2020) Building a just climate future for North Carolina. September 9^th^. Center for American Progress. Retrieved from https://www.americanprogress.org/issues/green/reports/2020/09/09/490114/building-just-climate-future-north-carolina/. Accessed 20 April 2021

[CR39] Coley JS, Hess DJ (2012). Green energy laws and Republican legislators in the United States. Energy Policy.

[CR40] Cook J (2017). Who’s regulating who? Analyzing fracking policy in Colorado, Wyoming, and Louisiana. Environ Pract.

[CR41] Culhane T, Hall G, Roberts JT (2021) Who delays climate action? Interest groups and coalitions in state legislative struggles in the United States. Energy Research and Social Science*.* Forthcoming

[CR42] Curtis S, Acuto M (2018). The foreign policy of cities. The RUSI Journal.

[CR43] Daniels DP, Krosnick JA, Tichy MP, Tompson T (2012) Public opinion on environmental policy in the United States. In Handbook of U.S. Environmental Policy, edited by M. Kraft and S. Kamieniecki (pgs. 461-486). New York: Oxford University Press

[CR44] Davis C (2012). The politics of fracking: regulating natural gas drilling practices in Colorado and Texas. Rev Policy Res.

[CR45] Douglas, Erin (2021). In oil-rich Texas, GOP lawmakers push bill to punish Wall Street for fossil fuel disinvestments. *The Texas Tribune,* 11 March, 2021. <https://www.texastribune.org/2021/03/11/texas-oil-gas-legislature-wall-street/>

[CR46] Dunlap RE, McCright AM (2010) Climate change denial: sources, actors and strategies. *Routledge Handbook of Climate Change and Society*

[CR47] Dunlap RE, McCright AA (2015) ‘Challenging climate change: the denial countermovement’, in Riley E. Dunlap and Robert J. (eds), Climate Change and Society New York: Oxford University Press, pp. 300–332

[CR48] Dunlap RE, Jacques PJ (2013). Climate change denial books and conservative think tanks: exploring the connection. Am Behav Sci.

[CR49] DSIRE (2021) Database of State Incentives for Renewables and Efficiency. http://www.dsireusa.org. Accessed 7 Oct 2021

[CR50] Egan PJ, Mullin M (2012). Turning personal experience into political attitudes: the effect of local weather on Americans’ perceptions about global warming. J Polit.

[CR51] Egan PH, Mullin E (2017). Climate change: US public opinion. Annu Rev Polit Sci.

[CR52] Energy Marketers Association of Rhode Island. 2021. U.S. fuel distributors, farmers launch joint climate initiative: project carbon freedom seeks to advance equitable, common-sense clean energy. https://warmth4ri.com/project-carbon-freedom/. Accessed 10 Jun 2021

[CR53] Erickson P, Lazarus M, Piggot G (2018). Limiting fossil fuel production as the next big step in climate policy. Nat Clim Chang.

[CR54] Fang L (2014) “Koch spends more than double top ten unions combined.” Republic Report, March 7

[CR55] Farrell J (2016). Corporate funding and ideological polarization about climate change. Proc Natl Acad Sci.

[CR56] Farrell J (2019) The growth of climate change misinformation in US philanthropy: evidence from natural language processing. Environmental Research Letters, 14

[CR57] Fiorino D (2006) The New Environmental Regulation. MIT Press, Cambridge, MA

[CR58] Fischlein M, Peterson TR, Stephens JC, Wilson EJ (2014). Which way does the wind blow? Analyzing the sub-national context for renewable energy deployment in the United States. Environmental Governance.

[CR59] Fisher D (2013). Understanding the relationship between subnational and national climate change politics in the United States: toward a theory of boomerang federalism. Eviron Plann C Gov Policy.

[CR60] Fowler L, Breen J (2013). The impact of political factors on states’ adoption of renewable portfolio standards. Electr J.

[CR61] Fownes JR, Yu C, Margolin, DB (2018) Twitter and climate change. Sociology Compass, 12(6)

[CR62] Galvin R, Healy N (2020). The Green New Deal in the United States: what it is and how to pay for it. Energy Res Soc Sci.

[CR63] Goldstein R, You HY (2017). Cities as lobbyists. Am J Pol Sci.

[CR64] Grant D, Bergsrand K, Running K (2014). Effectiveness of US state policies in reducing CO_2_ emissions from power plants. Nat Climate Change.

[CR65] Greenberg E, McKendry C (2021) Contested power: energy democracy and the repoliticization of electricity in the Western U.S. Energ Res Soc Sci

[CR66] Guber DL (2013). A cooling climate for change? Party polarization and the politics of global warming. Am Behav Sci.

[CR67] Gurney KR, Liang J, Roest G, Song Y, Mueller K, Lauvaux T (2021). Under-reporting of greenhouse gas emissions in US cities. Nat Commun.

[CR68] Hamilton LC (2011). Education, politics and opinions about climate change evidence for interaction effects. Clim Change.

[CR69] Hamilton LC, Stampone MD (2013) Blowin’in the wind: short-term weather and belief in anthropogenic climate change. Weather, Climate, and Society 5.2:112–119

[CR70] Hartman, K, Shields L (2021) State policies promoting hybrid and electric vehicles. https://www.ncsl.org/research/energy/state-electricvehicle-incentives-state-chart.aspx. Accessed 7 Oct 2021

[CR71] Healy N, Barry J (2017). Politicizing energy justice and energy system transitions: Fossil fuel divestment and a “just transition”. Energy Policy.

[CR72] Heiman MK, Soloman BD (2004). Power to the people: electric utility restructuring and the commitment to renewable energy”. Ann Assoc Am Geogr.

[CR73] Hempling S (2020). Regulating mergers and acquisitions of U.S. electric utilities: industry concentration and corporate complication. Elgar Publishing, Northampton, MA

[CR74] Hertel-Fernandez A (2014). Who passes business’s ‘model bills?’ policy capacity and corporate influence in U.S. state politics. Perspect Polit.

[CR75] Hertel-Fernandez A, Mildenberger M, Stokes LC (2019). Legislative staff and representation in Congress. Am Polit Sci Rev.

[CR76] Hertel-Fernandez A (2019) State capture: how conservative activists, big businesses, and wealthy donors reshaped the American states – and the nation. Oxford University Press, New York

[CR77] Hess DJ (2016). The politics of niche-regime conflicts: distributed solar energy in the United States. Environ Innov Soc Trans.

[CR78] Hess DJ (2018). Energy democracy and social movements: a multicoalition perspective on the politics of energy transitions. Energy Res Soc Sci.

[CR79] Hess DJ, Lee D (2020). Energy decentralization in California and New York: conflicts in the politics of shared solar and community choice. Renew Sust Energ Rev.

[CR80] Hess DJ, Mai QD, Brown KP (2016). Red states, green laws: ideology and renewable energy legislation in the United States. Energy Res Soc Sci.

[CR81] Hess DJ, Gentry H (2019) 100% renewable energy policies in U.S. cities: strategies, recommendations, and implementation challenges. Sustain: Sci Pract Policy 15:45–61

[CR82] Hess DJ (2012) Good green jobs in a global economy. MIT Press, Cambridge, MA

[CR83] Hess DJ (2014) When green became blue: Epistemic rift and the corralling of climate science. In Fields of knowledge: science, politics and publics in the neoliberal age. Emerald Group Publishing

[CR84] Hirsh RF (1999). Power loss: The origins of deregulation and restructuring in the American electric utility system*.* MIT Press, Cambridge, MA

[CR85] Homsy GC (2020). Capacity, sustainability, and the community benefits of municipal utility ownership in the United States. J Econ Policy Reform.

[CR86] Hornsey MJ, Harris EA, Bain PG, Fielding KS (2016). Meta-analyses of the determinants and outcomes of belief in climate change. Nat Climate Change.

[CR87] Howe PD, Mildenberger M, Marlon JR, Leiserowitz A (2015). Geographic variation in opinions on climate change at state and local scales in the USA. Nat Climate Change.

[CR88] Howarth C, Black R (2015). Local science and media engagement on climate change. Nat Clim Chang.

[CR89] Huber MT (2021) Still no shortcuts for climate change. *Catalyst*. 4

[CR90] Hughes S (2019) Repowering cities: Governing climate change mitigation in New York City, Los Angeles, and Toronto. Cornell University Press, Ithaca, NY

[CR91] IPCC 2014 *Climate Change 2014: Synthesis Report. Contribution of Working Groups I, II and III to the Fifth Assessment Report of the Intergovernmental Panel on Climate Change* ed R K Pachauri and L A Meyer (Geneva: IPCC)

[CR92] IRS (2021). Measuring lobbying activity: expenditure test. https://www.irs.gov/charities-non-profits/measuring-lobbying-activity-expenditure-test Accessed 4 May 2021

[CR93] Isser S (2015) Electricity restructuring in the United States. Cambridge University Press, New York

[CR94] Jacques PJ, Dunlap RE, Freeman M (2008). The organisation of denial: conservative think tanks and environmental skepticism. Env Polit.

[CR95] Karapin R (2020). Federalism as a double-edged sword: the slow energy transition in the United States. J Environ Dev.

[CR96] Karapin R (2016) Political opportunities for climate policy: California, New York, and the federal government. Cambridge University Press, New York

[CR97] Jansa JM, Hansen ER, Gray VH (2019). Copy and paste lawmaking: legislative professionalism and policy reinvention in the states.". Am Pol Res.

[CR98] Kern K (2019). Cities as leaders in EU multilevel climate governance: embedded upscaling of local experiments in Europe. Environmental Politics.

[CR99] Kelsey N, Meckling J (2018) Who wins in renewable energy? Evidence from Europe and the United States. Energy Res Soc Sci 37:65–73

[CR100] Konisky DM, Hughes L, Kaylor CH (2016). Extreme weather events and climate change concern. Clim Change.

[CR101] Konisky DM, Woods ND (2018) Environmental federalism and the Trump presidency: a preliminary assessment. Publius: The Journal of Federalism 48:345–371

[CR102] Krause RM, Hawkins C (2021) Implementing city sustainability: overcoming administrative silos to achieve functional collective action. Temple University Press, Philadelphia, PA

[CR103] Lee T, Jung HY (2018). Mapping city-to-city networks for climate change action: geographic bases, link modalities, functions, and activity. J Clean Prod.

[CR104] Legislative Energy Trends. https://www.ncsl.org/Portals/1/Documents/energy/2020-2021_Legislative_Energy_Trends_v04_35914.pdf. Accessed 7 Oct 2021

[CR105] Leiserowitz A, Maibach E, Roser-Renouf C, Smith N (2011) Global warming’s six Americas, May 2011. Yale University and George Mason University

[CR106] Leiserowitz A, Maibach E, Rosenthal S, Kotcher J, Ballew MT, Bergquist P, Gustafson A, Goldberg M, Wang X (2020) Politics and global warming, April 2020

[CR107] Lennon M (2017). Decolonizing energy: Black Lives Matter and technoscientific expertise amid solar transitions. Energy Res Soc Sci.

[CR108] Lubell M, Zahran S, Vedlitz A (2007). Collective action and citizen responses to global warming. Polit Behav.

[CR109] Marlon J, Howe P, Mildenberger M, Leiserowitz A, Wang X. Yale climate opinion maps 2020. https://climatecommunication.yale.edu/visualizations-data/ycom-us/. Accessed 1 May 2021

[CR110] May PJ, Koski C (2007) State environmental policies: analyzing green building mandates. Rev Policy Res 24(1):49–65

[CR111] Mayer J (2017) Dark money: the hidden history of the billionaires behind the rise of the radical right. Anchor Books

[CR112] Matisoff DC, Edwards J (2014). Kindred spirits or intergovernmental competition? The innovation and diffusion of energy policies in the American states (1990–2008). Environ Polit.

[CR113] McCarthy J (2016) A Worry about terror attacks in U.S. high, but not top concern. Gallup. http://www.gallup.com/poll/190253/worry-terror-attacks-high-not-top-concern.aspx. Accessed 15 Jun 2021

[CR114] McCright AM, Dunlap RE (2003). Defeating Kyoto: the conservative movement’s impact on US climate change policy. Soc Probl.

[CR115] McCright AM, Dunlap RE (2011). The politicization of climate change and polarization in the American public’s views of global warming, 2001–2010. Sociol Q.

[CR116] McCright AM, Dunlap RE, Marquart-Pyatt ST (2016). Political ideology and views about climate change in the European Union. Environmental Politics.

[CR117] McCright AM, Dunlap RE (2015) Challenging climate change. In Climate change and society: sociological perspectives 300–332

[CR118] Meckling J, Nahm J (2018). The power of process: state capacity and climate policy. Governance.

[CR119] Michaels D (2008). Doubt is their product: how industry’s assault on science threatens your health. Oxford University Press, New York

[CR120] Mikkelson GM, Avidan M, Conevska A, Etzion D (2021) Mutual reinforcement of academic reputation and fossil fuel divestment. Global Sustainability 4:e20

[CR121] Mildenberger M, Leiserowitz A (2017) Public opinion on climate change: is there an economy-environment tradeoff? Environ Politics 26:801–24

[CR122] Mildenberger M (2020) Carbon captured: how business and labor control climate politics. MIT Press, Cambridge, MA

[CR123] Miras NS, Rouse SM (2021) Partisan misalignment and the counter-partisan response: how national politics conditions majority party policy making in the American states. Br J Polit Sci 1–20

[CR124] Mooney CZ (2012). Explaining legislative leadership influence: simple collective action or conditional explanations?. Polit Res Q.

[CR125] Myers T, Nisbet MC, Maibach EW, Leiserowitz AA (2012). A public health frame arouses hopeful emotions about climate change. Clim Change.

[CR126] Nguyen Long LA, Krause RM (2020) Managing policy-making in the local climate governance landscape: the role of network administrative organizations and member cities. Public Admin. 1–17. 10.1111/padm.12684

[CR127] Open Secrets (2020a) Americans for Prosperity Action. https://www.opensecrets.org/outsidespending/detail.php?cycle=2020a&cmte=C00687103. Retrieved 6 Sept 2021

[CR128] Open Secrets (2020b) Americans for Prosperity Action. https://www.opensecrets.org/search/?q=Environmental+Organizations&cx=010677907462955562473. Retrieved 6 Sept 2021

[CR129] Oreskes N, Conway EM (2008) Challenging knowledge: how climate science became a victim of the Cold War. In: Proctor, R.N., Schiebinger, L. (Eds.), Agnotology: The Making and Unmaking of Ignorance. Stanford University Press pp. 55–89

[CR130] Piggot G (2018). The influence of social movements on policies that constrain fossil fuel supply. Climate Policy.

[CR131] Prechel H (2012). Corporate power and U.S. economic and environmental policy, 1978–2008. Camb J Reg Econ Soc.

[CR132] Prechel H (2021). Neoliberal Organizational and Political-Legal Arrangements and Greenhouse Gas Emissions in the U.S. Electrical Energy Sector. Sociol Q.

[CR133] Prechel H (2021a) Normalized financial wrongdoing. Stanford University Press, Stanford, CA

[CR134] Rabe BG (2004) Statehouse and greenhouse: the emerging politics of American climate change policy. Brookings Institution Press, Washington D.C

[CR135] Rabe BG (2007) Beyond Kyoto: climate change policy in multilevel governance systems. Governance: Int J Policy Admin Inst 20:423–444

[CR136] Rabe BG (2011) Contested Federalism and American Climate Policy. Publius: The Journal of Federalism 41:494–521

[CR137] Rabe BG (2013). Racing to the top, the bottom, or the middle of the pack? The evolving state government role in environmental protection. In N. Vig & M, Kraft (Eds.), *Environmental policy: New directions for the 21*^*st*^* century* (pp,30–53). CQ Press, Washington, D.C.

[CR138] Raymond L (2016). Reclaiming the atmospheric commons: the Regional Greenhouse Gas Initiative and a new model of emissions trading. MIT Press, Cambridge, MA

[CR139] Riverstone-Newell L (2017) The rise of state preemption laws in response to local policy innovation. Publius: The Journal of Federalism 47:403–425

[CR140] Romankiewicz J, Bottorff C, Stokes LC (2021) The dirty truth about utility climate pledges. Sierra Club. https://www.sierraclub.org/sites/www.sierraclub.org/files/blog/Final%20Greenwashing%20Report%20%281.22.2021%29.pdf>. Accessed 6 Apr 2021

[CR141] Schattschneider E (1960) The Semi sovereign people. Holt, Rinehart and Winston, New York

[CR142] Senate Assembly (2019) Relates to the New York state climate leadership and community protection act, Senate Bill S6599 A8429, 2019–2020 Legislative Session

[CR143] Shao W, Xian S, Keim BD, Goidel K, Lin N (2017). Understanding perceptions of changing hurricane strength along the US Gulf Coast. Int J Climatol.

[CR144] Shay LP (2020) Do term limits ‘limit’ the Speaker? Examining the effects of legislative term limits on state Speaker power. State Polit Policy Q 21(2):139–164

[CR145] Si Y, Stephens JC (2021) "Energy justice through solar: constructing and engaging low-income households." Frontiers in Sustainable Cities 3(20)

[CR146] Sierra Club. 2021. https://www.sierraclub.org/ready-for-100. Accessed 25 May 2021

[CR147] Slattery MC, Lantz E, Johnson BL (2011). State and local economic impacts from wind energy projects: Texas case study. Energy Policy.

[CR148] Smith KM, Koski C, Siddiki S (2021). Regulating net metering in the United States: a landscape overview of states’ net metering policies and outcomes.” The Electricity Journal 34(2)

[CR149] Stephens JC (forthcoming 2021). "Beyond climate isolationism: a necessary shift for climate justice." Current Opinion in Environmental Sustainability

[CR150] Stephens JC (2019). Energy democracy: redistributing power to the people through renewable transformation. Environment: Science and Policy for Sustainable Development 61:4–13

[CR151] Stephens JC (2020) Diversifying power: why we need antiracist, feminist leadership on climate and energy. Island Press, Washington D.C.

[CR152] Stephens J, et al. (2018) "The role of college and university faculty in the fossil fuel divestment movement." Elementa: Science of the Anthropocene 6(1):41

[CR153] Stokes L, Warshaw C (2017). Renewable energy policy design and framing influence public support in the United States. Nat Energy.

[CR154] Stokes LC (2020). Short circuiting policy. Oxford University Press, New York

[CR155] Stoutenborough JW, Bromley-Trujillo R, Vedlitz A (2014). Public support for climate change policy: consistency in the influence of values and attitudes over time and across specific policy alternatives. Rev Policy Res.

[CR156] Stuart D, Gunderson R, Petersen B (2020). The climate crisis as a catalyst for emancipatory transformation: an examination of the possible. Int Sociol.

[CR157] Squire P (2007) Measuring state legislative professionalism: The Squire Index revisited. State Polit Policy Q 7:211–227

[CR158] Tandoc EC Jr, Eng N (2017) Climate change communication on Facebook, Twitter and Sina Weibo. Oxford Research Encyclopedia of Climate Science, 1, pp. 603-615

[CR159] Tesler M (2018). Elite domination of public doubts about climate change (not evolution). Polit Commun.

[CR160] Thomson V (2014) Sophisticated interdependence in climate policy: federalism in the United States, Brazil and Germany. Anthem Press, New York

[CR161] Trachtman S (2020) What drives climate policy adoption in the U.S. states? Energy Policy 138

[CR162] Trinks A (2018). Fossil fuel divestment and portfolio performance. Ecol Econ.

[CR163] U.S. Energy Information Administration (2017) Investor-owned utilities served 72% of U.S. electricity customers in 2017. Today in Energy*.* <https://www.eia.gov/todayinenergy/detail.php?id=40913>. Accessed 6 Apr 2021

[CR164] U.S. Environmental Protection Agency (2018) Sources of greenhouse gas emissions. https://www.epa.gov/ghgemissions/sources-greenhouse-gas-emissions. Accessed 24 May 2021

[CR165] United States Climate Alliance (2019) U.S. Climate Alliance Governors Oppose Administration’s Withdrawal from the Paris Agreement. https://www.usclimatealliance.org/publications/pariswithdrawal . Accessed 25 May 2021

[CR166] United States Climate Alliance (2021) Further, faster, together fact sheet. https://static1.squarespace.com/static/5a4cfbfe18b27d4da21c9361/t/61b391a282d69906666f40fc/1639158179664/USCA_2021+Fact+Sheet+211208.pdf. Accessed 20 June 2021

[CR167] US Conference of Mayors (2014) “Successful city initiatives with energy efficiency and Conservation Block Grant (EECBG) Funding. (February 2014) http://www.usmayors.org/wp-content/uploads/2017/06/0227-report-eecbgsurvey.pdf. Accessed 25 May 2021

[CR168] van der Heijden J, Patterson J, Juhola S, Wolfram M (2019). Special section: advancing the role of cities in climate governance–promise, limits, politics. J Environ Planning Manage.

[CR169] Vasseur M (2014). Convergence and divergence in renewable energy policy among U.S. states from 1998 to 2011. Soc Forces.

[CR170] We are still in (2021) “Who’s in” https://www.wearestillin.com/signatories . Accessed 20 May 2021

[CR171] Wingfield B, Marcus M (2007) “America’s Greenest States.” http://www.forbes.com/2007/10/16/environment-energy-vermont-biz-beltway-cx_bw_mm_1017greenstates.html. Retrieved 25 Aug 2016

[CR172] Wolcott J (2007) “Rush to judgment” Vanity Fair. May 2007

[CR173] Woods ND (2021). The state of state environmental policy research: a thirty-year progress report. Rev Policy Res.

